# NF-κB Pathways in the Pathogenesis of Multiple Sclerosis and the Therapeutic Implications

**DOI:** 10.3389/fnmol.2016.00084

**Published:** 2016-09-15

**Authors:** Saskia M. Leibowitz, Jun Yan

**Affiliations:** UQ Centre for Clinical Research, The University of QueenslandBrisbane, QLD, Australia

**Keywords:** NF-κB, multiple sclerosis, IκB-α, IKK, signaling pathway

## Abstract

Nuclear factor kappa-light-chain-enhancer of activated B cells (NF-κB) signaling pathways are involved in cell immune responses, apoptosis and infections. In multiple sclerosis (MS), NF-κB pathways are changed, leading to increased levels of NF-κB activation in cells. This may indicate a key role for NF-κB in MS pathogenesis. NF-κB signaling is complex, with many elements involved in its activation and regulation. Interestingly, current MS treatments are found to be directly or indirectly linked to NF-κB pathways and act to adjust the innate and adaptive immune system in patients. In this review, we will first focus on the intricacies of NF-κB signaling, including the activating pathways and regulatory elements. Next, we will theorize about the role of NF-κB in MS pathogenesis, based on current research findings, and discuss some of the associated therapeutic implications. Lastly, we will review four new MS treatments which interrupt NF-κB pathways—fingolimod, teriflunomide, dimethyl fumarate (DMF) and laquinimod (LAQ)—and explain their mechanisms, and the possible strategy for MS treatments in the future.

## Introduction

Multiple sclerosis (MS) is a chronic, inflammatory, demyelinating disease of the human central nervous system (CNS; Pender and Greer, 2007). MS causes vision, motor and sensory problems, as well as a decline in cognitive, bladder and bowel function (Rudick et al., 1992; Yan and Greer, 2008).

MS pathogenesis has both genetic and environmental factors. Genes involved in immune function have been shown to confer risk to MS, for example, the MHC class II allele HLA-DRB1*15 (Miljković and Spasojević, 2013) Furthermore, IL-2, IL-7 and CTLA-4 genes have also been linked to MS (Miljković and Spasojević, 2013). Low vitamin D levels have also been flagged as a possible factor and may influence immune functioning (Miljković and Spasojević, 2013). Another molecule associated with immune functioning that has been implicated in MS pathogenesis is nuclear factor kappa-light-chain-enhancer of activated B cells (NF-κB).

Inflammatory and autoimmune diseases such as MS are associated with constitutive activation of NF-κB which leads to excessive expression of the effector molecules whose transcription relies on the NF-κB pathway (Li and Verma, 2002; Yamamoto and Gaynor, 2004). NF-κB acts on many immune cells, producing effects that increase inflammation, as seen in MS (Yan and Greer, 2008). NF-κB is crucial to the development, proliferation and survival of B and T lymphocytes and is involved in processes such as antibody class switching, CD4^+^ T cell differentiation and cytokine production (Yan and Greer, 2008). NF-κB also induces the production of inflammatory mediators by dendritic cells, enhances antigen processing and presentation in macrophages, and causes production of pro-inflammatory cytokines and neurotoxic mediators in microglia and astrocytes (Yan and Greer, 2008). It is clear that excessive expression of NF-κB could promote a pro-inflammatory milieu in which autoimmune diseases could develop.

## Overview of NF-κB Activation

The mammalian NF-κB family is highly conserved and comprises p65 (RelA), RelB, c-Rel, p50/p105 (NF-κB1) and p52/p100 (NF-κB2; Zheng et al., 2011). These proteins all possess a rel homology region (RHR) that consists of the N-terminal domain (NTD), dimerization domain (DD) and nuclear localization sequence (NLS; Huxford et al., 2011). These domains allow dimerization (through DD), DNA binding to target genes (through NTD and DD) and nuclear translocation (through NLS; Huxford et al., 2011). Alone, each member of the family is inactive in the cytoplasm in the cells. The proteins assemble in various combinations to form active homodimers and heterodimers (Huxford et al., 2011). The NF-κB dimers interact with specific sequence motifs known as κB sites on their target genes and activate transcription (Zheng et al., 2011). These κB sites show great variability, with each different dimer combination having a distinct DNA-binding site (Zheng et al., 2011).

The NF-κB proteins are tightly regulated by interactions with the inhibitor of κB (IκB) family. These family members possess an ankyrin repeat domain (ARD) that interacts with the RHR, preventing the transcription factor from performing the functions described previously (Zheng et al., 2011).

The IκB family consists of the classical IκBs (IκB-α, IκBβ and IκBɛ), the NF-κB precursors (p105 and p100) and nuclear IκBs (IκBζ, Bcl-3 and IκB_NS_; Malek et al., 1998, 2001; Zheng et al., 2011). The classical IκBs inhibit p65, RelB and c-Rel and bind dimers containing at least one p65 or c-Rel subunit (Zheng et al., 2011). In contrast, p105 and p100 actually contain an IκB-like domain that inhibits RHR (Zheng et al., 2011; Tao et al., 2014). The nuclear IκBs bind p50 or p52 homodimer (Zheng et al., 2011).

Certain stimuli, such as cytokines, chemokines, bacterial and viral products, free radicals and UV radiation, can trigger NF-κB activation (Yamamoto and Gaynor, 2004). There are at least two NF-κB activation pathways that have been described: the canonical and non-canonical pathways.

## The Canonical NF-κB Signaling Pathway

The NF-κB canonical pathway is involved in gene activity influencing cell biological processes in innate and adaptive immunity, inflammation, stress, lymphoid organogenesis and B cell development (Zhu et al., 2007). In the canonical pathway, cells are stimulated by factors such as TNF-α, IL-1β and LPS (Adhikari et al., 2007). Normally, NF-κB p65/p50 dimer is bound to IκB-α — the inhibitor of NF-κB—and exists in the cytoplasm in an inactive form (Phelps et al., 2000; Li and Verma, 2002). IκB proteins mask NLS on NF-κB subunits, causing the NF-κB:IκB complex to remain in the cytoplasm (Li and Verma, 2002).

**Figure 1:**
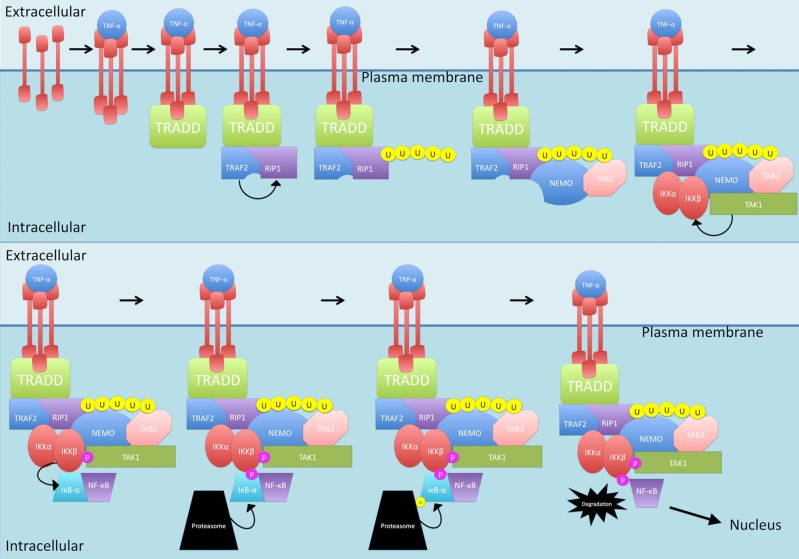
**The TNF-α-activated canonical nuclear factor kappa-light-chain-enhancer of activated B cells (NF-κB) signaling pathway.** TNF-α triggers TNF-R1 receptor trimerization. TNF receptor type 1-associated death domain protein (TRADD) is then recruited which, in turn, recruits TNF receptor-associated factor 2 (TRAF2) and receptor-interacting protein 1 (RIP1). TRAF2 mediates K63-linked polyubiquitination of RIP1 which allows recruitment of TAB2 and NF-κB essential modulator (NEMO). Following this, TAB2 recruits transforming growth factor beta-activated kinase 1 (TAK1) while NEMO and TRAF2 recruit the IKK complex. TAK1 phosphorylates IKKβ which then phosphorylates IκB-α. IκB-α is then ubiquitinated and degraded, releasing NF-κB and allowing translocation into the nucleus. P, phosphate; U, ubiquitin.

The canonical pathway can be initiated by TNF-α (see Figure 1). Interestingly, levels of this cytokine have been reported to be elevated in disease situations, including in MS (Rentzos et al., 1996; Ozenci et al., 2000). When TNF-α interacts with the TNF-R1 receptor, trimerization is initiated (Devin et al., 2000). This trimerization leads to recruitment of TNF receptor type 1-associated death domain protein (TRADD) which in turn recruits TNF receptor-associated factor 2 (TRAF2) and receptor-interacting protein 1 (RIP1; Devin et al., 2000). TRAF2 is an E3 ubiquitin ligase that then mediates K63-linked polyubiquitination of RIP1 (Adhikari et al., 2007; Spiegel and Milstien, 2011). Compared to K48-linked polyubiquitination which causes proteasomal degradation of target proteins, K63-linked polyubiquitination allows RIP1 to recruit proteins (Spiegel and Milstien, 2011). The polyubiquitinated RIP1 provides a scaffold for the binding of TAB2 and NF-κB essential modulator (NEMO), the regulatory unit of the IKK complex (Ea et al., 2006). TAB2 recruits Transforming growth factor beta-activated kinase 1 (TAK1) while NEMO and TRAF2 recruit the IKK complex, comprising the IKKα and IKKβ subunits. TAK1, which is rapidly activated after stimulation of cells with TNF-α (as well as IL-1β, TLR and TCR) now in close proximity with IKK, phosphorylates IKKβ, activating it (Wang et al., 2001; Ea et al., 2006). IKKβ then can phosphorylate IκB-α which is then ubiquitinated and degraded, releasing NF-κB from the binding and allowing it to become the activated form (Devin et al., 2000). NF-κB then can translocate into the nucleus and bind to the promoter of genes to start gene transcription.

**Figure 2:**
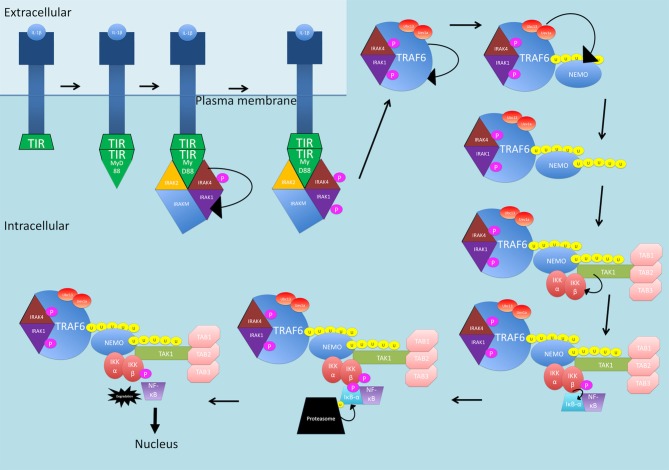
**The IL-1β-activated canonical NF-κB signaling pathway.** The receptor is activated by IL-1β and recruits MyD88 through homophilic interactions between toll/interleukin-1 receptor (TIR) domains. MyD88 interacts with IRAK1, IRAK2, IRAKM and IRAK4. IRAK4 is phosphorylated and then phosphorylates and activates IRAK1. IRAK1 and IRAK4 interact with TRAF6 which self-ubiquitinates and also ubiquitinates NEMO through Ubc13-Uev1a, in complex with TRAF6. NEMO and TRAF6 recruit TAK1 in complex with TABs1–3. TAK1, activates IKKβ, which then phosphorylates IκB-α. IκB-α is then ubiquitinated and degraded, releasing NF-κB and allowing translocation into the nucleus. P, phosphate; U, ubiquitin.

IL-1β activates the NF-κB canonical pathway through a MyD88-dependent pathway (see Figure 2). IL-1 receptors and TLRs all possess a Toll/interleukin-1 receptor (TIR) domain (Kawai and Akira, 2007). On receptor activation, through homophilic interaction, the receptors recruit adaptor proteins that also possess TIR domains (Kawai and Akira, 2007). MyD88 comprises a TIR domain and is recruited. From here, MyD88 interacts with IRAK1, IRAK2, IRAKM and IRAK4 (Kawai and Akira, 2007). IRAK4 is activated first and phosphorylates and activates IRAK1. IRAKM prevents the dissociation of IRAK1 and IRAK4 from MyD88 (Kawai and Akira, 2007). The function of IRAK2 is unknown. After phosphorylation, IRAK1 and IRAK4 interact with TRAF6 which causes self-ubiquitination as well as ubiquitination of NEMO through Ubc13**-**Uev1a which are in complex with TRAF6 (Kawai and Akira, 2007). Ubiquitinated NEMO and TRAF6 then recruit TAK1 in complex with TABs1–3. The MAPK pathway involving ERK is activated and this, along with TAK1, activates IKKβ, leading to NF-κB release (Kawai and Akira, 2007).

**Figure 3:**
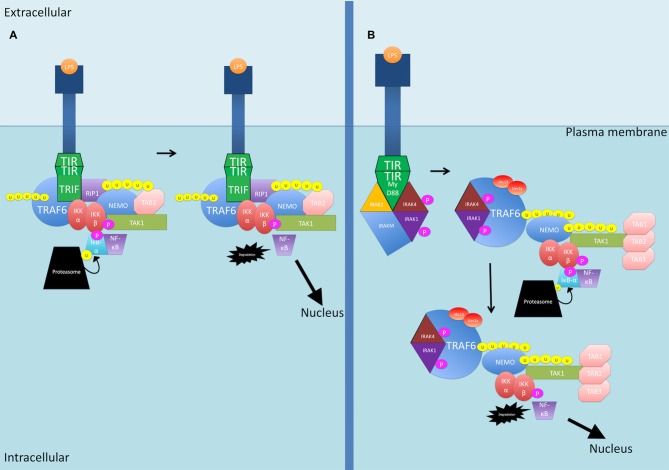
**The LPS-activated canonical NF-κB signaling pathways. (A)** TIR-domain-containing adapter-inducing interferon-β (TRIF) is recruited to the TLR4 receptor. TRAF6 and RIP1 are then recruited. TRAF6 is polyubiquitinated and together with RIP1, the two proteins activate TAK1. **(B)** The MyD88 pathway as described previously for IL-1β. P, phosphate; U, ubiquitin.

LPS binding to TLRs activates two primary pathways downstream of TLR4 (see Figure 3). In the first pathway, TIR-domain-containing adapter-inducing interferon-β (TRIF) is recruited, and then recruits TRAF6 and RIP1 to the receptor (Wertz and Dixit, 2010). The polyubiquitination of TRAF6 and RIP1 facilitates TAK1 activation as described previously (Wertz and Dixit, 2010). The second pathway is the MyD88 pathway as described previously for IL-1β (Wertz and Dixit, 2010).

**Figure 4:**
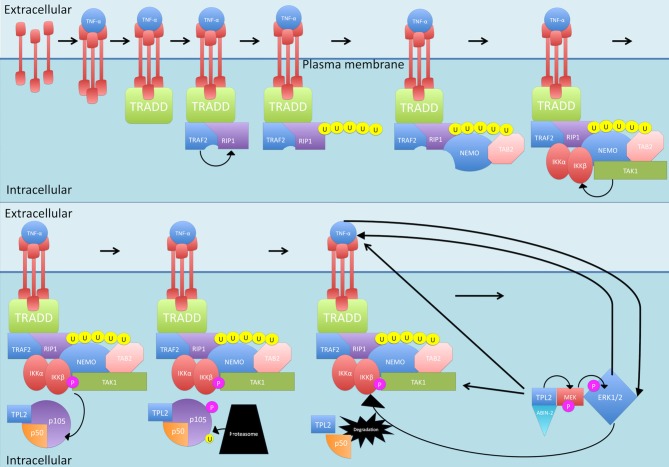
**Canonical activation of p50.** Once activated by normal canonical signaling, IKKβ phosphorylates NF-κB subunit p105, which is the precursor of NF-κB subunit p50. Phosphorylation of p105 generates binding sites for ubiquitin ligases that then target p105 for degradation. p105 also inhibits tumor progression locus 2 (TPL2) by binding with it. Once p105 is degraded, TPL2 is released and stabilized by binding to MEK and ABIN-2. TPL2 then phosphorylates MEK which phosphorylates ERK, ERK1/2 leads to increased TNF-α production and can activate IKK. P, phosphate; U, ubiquitin.

Another protein phosphorylated by IKKβ is the NF-κB subunit p105, which is the precursor of NF-κB subunit p50 (see Figure 4). p105 is called *NF-KB1* (Belich et al., 1999). p105, like the other IκB family members, possessing an ARD, acts as an inhibitor of not just p50 but also c-Rel and RelA, retaining them in the cytoplasm and thus blocking their transcription functions (Lang et al., 2003). In order to release the inhibition, p105 must be proteolysed (Belich et al., 1999; Gantke et al., 2011). IKKβ phosphorylation of p105 generates binding sites for ubiquitin ligases that then target p105 for degradation (Lang et al., 2003) While there is a basal level of constitutive, ubiquitin-independent processing that occurs, this signal-dependent degradation of p105 accelerates the process (Lang et al., 2003; Moorthy et al., 2006). As well as inhibiting NF-κB translocation, p105 also exerts inhibitory effects on tumor progression locus 2 (TPL2), a MAP 3 kinase that is activated by TLR and TNF-αR stimulation (Gantke et al., 2011). In steady state conditions, the entire pool of TPL2 is associated with p105 but only a third of the p105 pool is occupied by TPL2 (DeCicco-Skinner, 2012). The target of TPL2 phosphorylation is MEK which once activated, can phosphorylate ERK (see Figure 4; Eliopoulos et al., 2002). This is significant as ERK1/2 leads to increased TNF-α production, increasing TNF-α-induced NF-κB production (van der Bruggen et al., 1999). TNF-α itself can also activate ERK1/2 (Lebman and Spiegel, 2008). In addition, ERK1/2 is suggested to play a role in IKK activation, which also results in NF-κB production (Chen and Lin, 2001).

Interestingly, p105 does not inhibit the catalytic activity of TPL2, so it is possible that other targets of this molecule are being phosphorylated, but being in complex with p105 somehow prevents phosphorylation of MEK (Gantke et al., 2011). Binding of TPL2 to MEK as well as the ubiquitin-binding protein ABIN-2 offers stability to TPL2 (Gantke et al., 2011). The mechanism required to release the inhibitory effect of p105 on TPL2 is the same as that required to lift the inhibition on p50—proteolysis following phosphorylation by IKK (Belich et al., 1999; Gantke et al., 2011). Once p105 is degraded by the proteasome, TPL2 can phosphorylate MEK (van der Bruggen et al., 1999; Gantke et al., 2011).

TPL2 can also cause the production of TNF-α during inflammatory responses (Gantke et al., 2011). In fact, the TPL2/ERK pathway has been found to promote transport of the TNF-α mRNA from the nucleus to the cytoplasm (Dumitru et al., 2000). It has been found that blocking this pathway is sufficient to inhibit the induction of TNF-α (Dumitru et al., 2000).

Other influences of TPL2 on the canonical pathway include its interaction with TAK1, mediating responses to cytokines TNFα or IL-1, through direct phosphorylation of IKK, leading to its activation (Freudlsperger et al., 2013).

## Non-Canonical NF-κB Signaling Pathway

**Figure 5:**
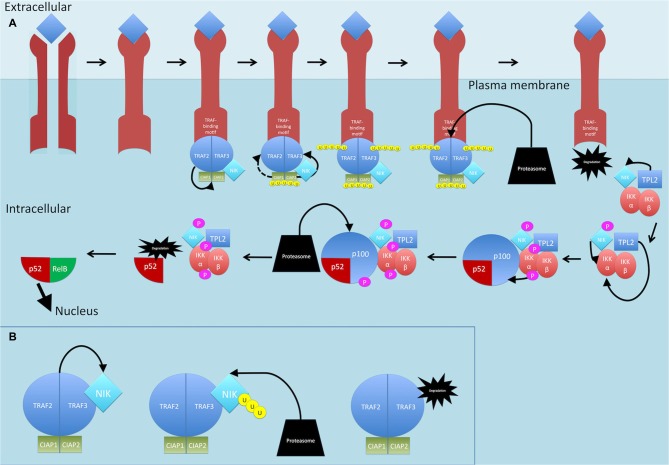
**Non-canonical NF-κB signaling pathway. (A)** On ligand binding, receptors crosslink and the TRAF2:TRAF3:CIAP1/2 complex is recruited to the receptors. TRAF2 ubiquitinates cIAP1 and cIAP2, which then ubiquitinate TRAF3 (and TRAF2 to some extent) and stimulate its rapid degradation. NF-κB-inducing kinase (NIK) is released. TPL2 physically assembles with IKK and NIK and phosphorylates NIK which then phosphorylates and activates IKKα. TPL2 also activates IKKα. IKKα phosphorylates p100. Once phosphorylated, p100 is degraded by the proteasome and p52 is released and can dimerize and translocate to the nucleus. **(B)** Dimerization of TRAF3 with TRAF2 allows recruitment of the CIAPs1/2 ubiquitin ligases, Normally, NIK is bound by this complex and targeted for constant ubiquitination and proteasomal degradation. P, phosphate; U, ubiquitin.

Activation of a subset of TNFR superfamily members including BAFFR, CD40, LTbR, RANK and TNFR2, leads to activation of NF-κB through the non-canonical pathway (see Figure 5; Sun, 2011). Activation of these receptors all share a common convergence on the activation of NF-κB-inducing kinase (NIK; Sun, 2011). Normally, NIK is bound by TRAF3 which targets it for constant ubiquitination and proteasomal degradation (Sun, 2011). This occurs through the dimerization of TRAF3, the adaptor molecule, with TRAF2, which can then allow recruitment of the CIAP1/2 ubiquitin ligases (Sun, 2011; Ersing et al., 2013). This complex ubiquitinates NIK for degradation. On ligand binding, receptors crosslink and the TRAF2:TRAF3:CIAP1/2 complex is recruited via the association between TRAF2 and TRAF3 and the TRAF-binding motif on the receptors (Sun, 2011) TRAF2 then ubiquitinates cIAP1 and cIAP2, which then ubiquitinate TRAF3 (and TRAF2 to some extent) and stimulate its rapid degradation (Vallabhapurapu et al., 2008; Ersing et al., 2013). NIK, now stabilized, reaches a threshold concentration and auto-activates its kinase activity (Ersing et al., 2013). NIK then phosphorylates and activates IKKα (Sun, 2011). Unlike the canonical pathway where IKKβ phosphorylates IκB-α, IKKα instead phosphorylates the NF-κB2 protein (Sun, 2011). The NF-κB2 gene, *NF-κB2*, encodes a precursor protein p100 from which p52 is derived (Sun, 2011). NF-κB p100 possesses an ARD that masks the NLS on p52. As mentioned, activation of TNFR superfamily members culminates in activation of NIK, which activates IKKα. It is proposed that formation of a NIK/IKKα/p100 complex may be critical for subsequent p100 phosphorylation by IKKα (Sun, 2011). Once phosphorylated, p100 is degraded by the proteasome and p52 can dimerize and translocate to the nucleus.

TPL2 is thought to play a role in the non-canonical pathway too. The mechanism by which this occurs is thought to involve IKK and NIK. TPL2 physically assembles with both these proteins and TPL2, through its interaction with NIK, activates NIK through phosphorylation (Lin et al., 1999; Eliopoulos et al., 2002). In addition, studies show that ectopic expression of TPL2 activates IKKα, meaning TPL2 is one of three MAP3Ks that can induce NF-κB through the IKKs, with the other two being MEKK1 and NIK (Lin et al., 1999).

## Defective NF-κB Signaling Pathways in MS

Many studies have found that NF-κB is activated in the brain tissue of patients with MS. In actively demyelinating plaques, there was increased nuclear localization of NF-κB subunits in microglia, a subset of hypertrophic astrocytes and lymphocytes (Gveric et al., 1998). Another study showed that in active MS lesions, there was upregulation of nuclear NF-κB in a large proportion of oligodendrocytes located at the edge of active lesions and in microglia throughout plaques but not in healthy white matter or silent MS plaques (Bonetti et al., 1999). Microarray analysis of MS brain tissue has also identified upregulation of NF-κB itself as well as genes related to NF-κB (Lock et al., 2002). One large study found that the genes encoding NF-κB inhibitors exhibited significant sequence variations (Miterski et al., 2002). In particular, they found one predisposing allele in an *IKBL* gene and a protective allele in the promoter of the IκB-α gene, *NFKBIA*, which was decreased in frequency in primary progressive MS (Miterski et al., 2002).

Variants have been identified within the NF-κB signaling pathway in subjects with autoimmune diseases through genome-wide association studies. In MS patients, variants near genes involved in NF-κB signaling have been found and are functional, potentially due to location in regulatory elements (Housley et al., 2015). One such variant, proximal to *NFKB1*, increased expression by twenty-fold (Housley et al., 2015). Another variant was found in an intron of the *TNFR1* gene, which led to increased NF-κB signaling after TNF-α stimulation (Housley et al., 2015). Both variants caused decreased expression of the negative regulators of NF-κB, particularly IκB-α, through degradation (Housley et al., 2015).

## NF-κB in MS Pathogenesis

### The Role of Epstein–Barr Virus in NF-κB-related Pathogenesis

NF-κB is associated with the pathogenesis of Epstein–Barr virus (EBV)-derived MS. Studies have shown the crucial role of EBV in the development of MS, with large studies showing that MS patients are almost all seropositive for EBV (Lünemann et al., 2007). There is a theory of molecular mimicry between EBV- and CNS-derived epitopes (Lünemann et al., 2007). EBV preferentially infects and transforms B lymphocytes by inducing activation and continuous proliferation of these cells (Lünemann et al., 2007). The transformed cells carry the viral genome and express nine latent proteins (Allday, 2009). One of these is LMP1, a membrane-bound signaling molecule (Allday, 2009).

LMP1 has been found to constitutively activate NF-κB through multiple mechanisms (Gewurz et al., 2011). First, LMP1 is an activator of the canonical pathway. LMP1 first activates TRAF6 through interactions with adaptor proteins (Ersing et al., 2013). A study suggests that the adaptor may be TRADD which then recruits a TRAF2:TRAF6 heterodimer to the complex (Schultheiss et al., 2001). TRAF6 then autoubiquitinates, recruiting TAB2 and TAB3 and thus activating TAK1 as per usual canonical signaling (Ersing et al., 2013). LMP1 also regulates expression of target genes, and canonical NF-κB is a critical component of this (Gewurz et al., 2011). LMP1 also activates the non-canonical pathway. LMP1 recruits TRAFs 1, 2, 3 and 5 (Ersing et al., 2013). While the mechanism remains to be elucidated, NIK is central to LMP1 induction on NF-κB activity. It is probably through phosphorylation of NIK that LMP1 triggers activation of IKKα, leading to phosphorylation of p100 (Ersing et al., 2013).

LMP1 mimics CD40 leading to constitutive activation of growth and survival pathways (Allday, 2009; Greenfeld et al., 2015). CD40 is a member of the TNFR family and has a motif to which TRAF2 can bind (Schultheiss et al., 2001). LMP1 has been shown to induce TRAF1 which heterodimerizes with TRAF2—LMP1 cannot mediate NF-κB activation without TRAF2 (Devergne et al., 1996). In addition to the intracellular signaling of LMP1, this protein can also be released in exosomes to act in the microenvironment (Lassmann et al., 2011). LMP1 has also been found to promote the activation of TPL2 which has a major influence on NF-κB signaling, as discussed previously (Eliopoulos et al., 2002).

After primary EBV infection, the expression of most, if not all, of the EBV latent proteins is down-regulated and a lifelong reservoir of latently infected memory B cells exists (Lassmann et al., 2011). These latent cells can be reactivated by certain stimuli, including local inflammatory cytokines like TNF, IL6 and CXCL13 (Lassmann et al., 2011). However, the persistent antigen presence of EBV-infected B-blasts triggers a robust T cell response that controls the events of reactivation (Hislop et al., 2005; Lünemann et al., 2007; Lassmann et al., 2011). The mechanism may involve LMP1 as it can act on T cells and studies have shown the responses are particularly T_H_1 polarized leading to IFN-γ, TNF-α and IL-2 secretion (Sohn et al., 2015).

A recent study has found that in MS, there is a decreased CD8^+^ T cell response to EBV (Pender et al., 2015). This was proposed to be the result of a primary quantitative deficiency in CD8^+^ T cells that may have a genetic background, as well as superimposed T cell exhaustion from decreased CD4^+^ T cell help (Pender et al., 2009, 2015). The deficiency in T cell immunity to EBV-infected cells may allow accumulation of autoreactive EBV-infected cells in the CNS and subsequent development of MS (Pender et al., 2009).

As LMP1 causes constitutive NF-κB production, it would be interesting to see if NF-κB has an effect on T cell function. One study demonstrated that constitutive activation of IKKβ, leading to constant production of NF-κB, as occurs in certain chronic viral infections, caused T cells to be less responsive to stimulation, triggered apoptosis and promoted autoimmunity (Krishna et al., 2012). They observed that these T cells resembled exhausted cells but were still able to produce cytokines (Krishna et al., 2012). Exhausted T cells are dysfunctional cells that arise during many chronic infections, and are characterized by poor effector function and subsequent deletion of antigen-specific T cells (Wherry, 2011). Therefore, the link between EBV and MS may involve NF-κB production through LMP1.

### NF-κB-related Immune Responses in MS and its Animal Model

Experimental autoimmune encephalomyelitis (EAE) is an acute or chronic relapsing demyelinating disease that is used as an experimental model of MS (Martin et al., 1995). It is induced in susceptible animal strains by injection of myelin or myelin components as well as adjuvants (Martin et al., 1995). Subsequently, an autoimmune response ensues, mediated by encephalitogenic T cells and the resultant demyelinating lesions resemble those seen in MS (Martin et al., 1995). Like MS, the pathogenesis of EAE is dependent on the activation of pro-inflammatory mediators, many of which are under the control of NF-κB (Pahan and Schmid, 2000). Hence, NF-κB has been a major focus of studies into EAE pathogenesis (Xie et al., 1994; Pahan and Schmid, 2000). The DNA-binding activity of NF-κB, specifically the RelA/p65 and p50 subunits, was induced in EAE rat spinal cords only, with NF-κB activation correlating with disease activity (Pahan and Schmid, 2000).

#### T_H_1 Responses in MS and EAE

NF-κB is known to be involved in T_H_1 responses and T_H_1 cells are involved in MS and EAE pathogenesis (Mazzeo et al., 1998; Becher and Segal, 2011). Serum levels of IL-12 were shown to be higher in patients with secondary progressive MS compared with controls and patients with other neurological diseases (Nicoletti et al., 1996). IL-12 levels were also higher in progressive MS compared with relapsing-remitting MS (Balashov et al., 1997). IL-12 is responsible for the raised IFN-γ secretion known to exacerbate MS, unlike in EAE, where IL-12 but not IFN-γ exacerbated EAE (Panitch et al., 1987; Balashov et al., 1997; Leonard et al., 1997; Segal et al., 1998).

T cells from MS patients induce IL-12 secretion from APCs through a CD40-dependent mechanism that is initiated by TCR engagement (Balashov et al., 1997). Subsequent signaling creates a chronic activation of T_H_1 responses (Balashov et al., 1997). Like IL-12 levels, which are higher in progressive MS, CD40 ligand-dependent T_H_1 activation occurred in the progressive but not relapsing–remitting form of MS (Balashov et al., 1997). As has been discussed, CD40 can activate the canonical and non-canonical NF-κB pathways. Furthermore, there is evidence to suggest that NF-κB is involved in IL-12 /p40 activation and IL-12 signaling (Murphy et al., 1995; Grohmann et al., 1998).

IL-18 augments the T_H_1 response as it can increase IFN-γ expression in T cells and NK cells (Losy and Niezgoda, 2001; Karni et al., 2002). Antibodies against IL-18 block EAE development (Wildbaum et al., 1998). In MS patients, IL-18 levels have been found to be increased in both serum and CSF with levels being higher in secondary progressive MS compared with relapsing-remitting MS, and higher in acute exacerbations compared with stable disease (Losy and Niezgoda, 2001; Nicoletti et al., 2001). IL-18 depends on NF-κB for signaling through MyD88 activation (Weinstock et al., 2003; Alboni et al., 2010). NF-κB has also been shown to be involved in IFN-γ expression, possibly through IL-12 and IL-18 (Sica et al., 1997).

IL-12 p70 is a heterodimer comprising p35 and p40 subunits. Deficiencies in either p35 or p40 prevent production of IL-12 p70, but mice deficient in only p35 are still highly susceptible to EAE (Becher and Segal, 2011). p40 knock-outs are resistant to EAE (Segal et al., 1998). Interestingly, the p40 subunit combines with a different factor, p19, to form IL-23 (Oppmann et al., 2000). Moreover, it has been found that mice deficient in p19 (deficient in IL-23) and p40 (deficient in IL-12 and IL-23) but not those deficient in p35 (deficient in IL-12) were resistant to EAE (Cua et al., 2003). Therefore, it was concluded that IL-23, not IL-12 is the critical factor in EAE development (Cua et al., 2003).

#### T_H_17 Responses in MS and EAE

IL-23 activates macrophage production of pro-inflammatory cytokines like IL-1 and TNF, cytokines which are known to activate the NF-κB signaling pathway (Cua et al., 2003). IL-23 also induces T_H_17 cells (Sun et al., 2013). NF-κB is involved in the induction of IL-23 in DCs and macrophages and has been shown to be required for T_H_17 development (Cho et al., 2006; Yang et al., 2008; Sun et al., 2013). T_H_17 cells have a characteristic cytokine profile, comprising IL-17, IL-21, IL-22 and GM-CSF. This cellular subset has been implicated in autoimmune inflammatory diseases such as MS. Patients with MS have higher percentages of T_H_17 cells, higher expression of IL-17 in cells near MS lesions and genetic linkage studies have shown the IL-17 and IL-17R genes to be of interest in MS (Matusevicius et al., 1999; Tzartos et al., 2008; Durelli et al., 2009; Muls et al., 2012). EAE, like MS, is largely driven by a T_H_17 response.

T_H_17 differentiation involves the retinoid-related orphan receptor-γ (Rorg or Rorc) which is under control of c-Rel and RelA (Ruan et al., 2011). Mice deficient in these NF-κB subunits have compromised T_H_17 differentiation and responses (Yang et al., 2008; Ruan et al., 2011). While RORγt-deficient mice have marked T_H_17 impairment, deficiency does not completely inhibit EAE development, while knock-out of RORα together with RORγt, globally impaired T_H_17 responses with complete protection from EAE (Yang et al., 2008).

IL-1β can also induce T_H_17 cells. In MS patients, the expression of IL-1β, IL-1 receptor accessory protein and IL-1 receptor antagonist (IL-1Ra) are increased in CSF (Dujmovic et al., 2009). Increased expression of IL-1Ra may seem counterintuitive as this molecule inhibits IL-1 functions, but it has been hypothesized to be part of a defense mechanism that may be involved in MS remissions (Dujmovic et al., 2009). Indeed, IL-1Ra administration leads to milder EAE signs than controls (Badovinac et al., 1998). The ability of IL-1 to promote IL-17 production by T cells has been shown to be dependent on NF-κB signaling (Sutton et al., 2006).

To understand how T_H_17 cells mediate EAE, it is important to elucidate the role of the effector cytokines produced by this cellular population. IL-17 causes chemokine and pro-inflammatory cytokine expression in astrocytes, which leads to leukocyte recruitment during the induction of CNS inflammation (Xiao et al., 2014). IL-17 mediates much of this pro-inflammatory signaling through up-regulation of NF-κB. IL-17 stimulation leads to the dissociation of TPL2 from p105, allowing it to associate with TAK and influence NF-κB production (Xiao et al., 2014). Indeed, TPL2 has been shown to be a crucial mediator of EAE (Xiao et al., 2014). In addition, Act1 is an adaptor molecule that recruits TRAF6 to the IL-17 receptor (Qu et al., 2012). Act1 mediates ubiquitination of TRAF6 which subsequently autoubiquitinates. It is then suggested that TRAF6 follows the pathway as in LPS and IL-1β signaling to generate NF-κB via the canonical pathway (Sønder et al., 2011). The onset and severity of EAE was greatly reduced in Act1-deficient mice (Kang et al., 2010).

However, while development of EAE is suppressed in IL-17-deficient mice, with impaired T cell sensitization against myelin antigens, it is not completely abolished (Komiyama et al., 2006). Therefore, while both IL-17A and IL-17F are highly expressed by encephalitogenic T cells, they may only marginally contribute to EAE development (Haak et al., 2009). Hence, IL-23 promotes EAE by IL-17 independent, as well as dependent, pathways (Becher and Segal, 2011). Therefore, T_H_17 cells must produce some other mediator of EAE.

T_H_17 cells also produce IL-21 and IL-22 (Becher and Segal, 2011). However, EAE can develop in the absence of IL-21 or IL-21R (Sonderegger et al., 2008). Furthermore, IL-22 does not appear to be directly involved in EAE (Kreymborg et al., 2007).

#### The Role of GM-CSF in MS and EAE

GM-CSF is the only known T_H_17 cytokine that is essential for EAE as mice deficient in GM-CSF are resistant to EAE, with decreased immune cell infiltration of the CNS (McQualter et al., 2001; Becher and Segal, 2011). IL-23 stimulation and RORγt drive expression of GM-CSF in T cells (Codarri et al., 2011). It is proposed that RORγt only partially protects from EAE as high homology of binding motifs shared with RORα allow it to compensate for the absence of RORγt in GM-CSF expression (El-Behi et al., 2011). Therefore, it follows that the double knockout of RORγt and RORα would perturb GM-CSF expression more fully, leading to complete protection from EAE. Both T_H_1 and T_H_17 subsets share the ability to up-regulate GM-CSF (Codarri et al., 2011; Grifka-Walk et al., 2015; McWilliams et al., 2015).

GM-CSF is crucial for the development of inflammatory demyelinating lesions and for controlling migration and proliferation of leukocytes within the CNS (McQualter et al., 2001). GM-CSF production by T cells is greater in untreated MS patients than healthy controls and IFN-β-treated MS patients, suggesting that GM-CSF contributes to MS pathogenesis too (Parajuli et al., 2012). GM-CSF has been shown to induce IL-23 in APCs which then induces GM-CSF expression by T_H_17 cells (Ponomarev et al., 2007; El-Behi et al., 2011).

p52 and c-Rel have been implicated in the induction of GM-CSF expression (Yu et al., 2014). It is proposed that it is through the IL1R/MyD88/NF-κB pathway that GM-CSF secretion by T cells and subsequent EAE are induced (Sutton et al., 2006). Additionally, after it is activated, GM-CSF has been shown to activate IKKβ, leading to IκB degradation and further activation of NF-κB (Ebner et al., 2003). Moreover, GM-CSF has been shown to increase production of IL-1β, IL-6, TNF-α and NO by up-regulating NF-κB (Parajuli et al., 2012). GM-CSF also up-regulates ERK1/2 (Parajuli et al., 2012). Therefore, this highlights that the pathogenic role of GM-CSF may relate to NF-κB activation.

## The Role of NF-κB Inhibitors in Disease Pathogenesis

Delving further into the control of NF-κB signaling in T_H_17 responses requires discussion of the atypical IκB proteins, a subset that includes Iκ*B*ζ, IκB_NS_ and BCL-3. The genes encoding these proteins are actually regulated by NF-κB and both positively and negatively modulate transcription by binding to NF-κB transcription factors in the nucleus (Ghosh and Hayden, 2008; Mankan et al., 2009; Schuster et al., 2012). The significance of these proteins is that they add an additional layer of control to NF-κB signaling. It is proposed that, depending on the inducing receptor, different IκB family members may be activated and may control NF-κB binding at specific gene promoters, leading to differential gene expression (Touma et al., 2007).

Of all the helper T cell subsets, Iκ*B*ζ is most highly expressed in T_H_17 cells and is induced by IL-17 (Okamoto et al., 2010; Sønder et al., 2011). Iκ*B*ζ up-regulates the transcription of key inflammatory mediators including IL-6, GM-CSF, G-CSF, IL-12p40, and IL-17, many of which are involved in T_H_17 responses (Hildebrand et al., 2013). Iκ*B*ζ recruitment to the regulatory region of the IL-17 gene is dependent on the ROR nuclear receptors and Iκ*B*ζ and the ROR nuclear receptors act synergistically to increase IL-17 expression (Okamoto et al., 2010). Iκ*B*ζ is a pro-inflammatory mediator and positively regulates specific NF-κB-dependent genes after LPS and IL-1 signaling, through the MyD88-dependent signaling pathways (Ghosh and Hayden, 2008). It associates with p50 homodimers that are bound to the IL-6 promoter, leading to IL-6 expression, a process that is abrogated in deficient mice (Yamamoto et al., 2004; Ghosh and Hayden, 2008).

Like the ROR knock-out mice, Iκ*B*ζ-deficient mice are also resistant to T_H_17-dependent EAE (Okamoto et al., 2010). This is possibly due to reduction in GM-CSF production, seeing as Iκ*B*ζ is involved in GM-CSF transcription. Furthermore, Iκ*B*ζ-deficient mice show decreased IL-17 production in the spleen and lymph nodes, indicating a possible defect in T_H_17 development (Okamoto et al., 2010). This results in a low sensitivity to EAE with almost no neuronal deficit (Okamoto et al., 2010).

IκB_NS_ is expressed during thymic natural T regulatory (Treg) cell development, and is involved in Forkhead box P3 positive (Foxp3) Treg induction through interactions with p50 and c-Rel and upon TGF-β treatment (Schuster et al., 2012). Foxp3 Treg cells are involved in maintaining peripheral tolerance (Yamamoto et al., 2004). One study found that mice deficient in IκB_NS_ had a 50% reduction of mature Treg cells (Schuster et al., 2012). In contrast, Iκ*B*ζ does not affect Treg cell development (Okamoto et al., 2010). Interestingly, even though Treg numbers were severely reduced in IκB_NS_-deficient mice, there were no signs of spontaneous autoimmunity (Schuster et al., 2012). This points towards potential involvement of IκB_NS_ in the activation, proliferation, or cytokine production of pro-inflammatory effector T cells (Yamamoto et al., 2004). Indeed, IκB_NS_ is involved in T cell effector function, with one study highlighting that knockout of IκB_NS_ led to reduced IL-2 and IFN-γ production after TCR triggering in CD8^+^ T cells (Touma et al., 2007). Another study showed strongly impaired T_H_17 responses, suggesting that IκB_NS_, like Iκ*B*ζ, is also required for T_H_17 differentiation and function (Annemann et al., 2015). Therefore, IκB_NS_ acts differentially in Tregs and effector T cells as in CD4^+^ T_H_ cells it regulates proliferation and effector cytokine production (Annemann et al., 2015). Furthermore, another group concluded that IκB_NS_-deficient mice had decreased expression of IL-17-related genes as well as RORγt in response to TGF-β and IL-6 stimulation (Kobayashi et al., 2014). Therefore, T_H_17 development was impacted and the deficient mice were resistant to developing EAE (Kobayashi et al., 2014).

## Therapeutic Implication of NF-κB Inhibitors in MS and EAE

Inhibitors of NF-κB can be of multiple classes. Inhibitors upstream of the IKK complex include: anti-TNF-α antibodies or TNF receptor blockers; TRAF2 and TRAF6 mutants; and MEKK1 and NIK mutants (Gilmore and Herscovitch, 2006). Anti-TNF-α therapy has been shown to cause exacerbations of MS (Titelbaum et al., 2005). Studies have found that TNF-α exerts both pro-inflammatory and potent immunosuppressive effects, explaining exacerbation of MS following anti-TNF treatment (Kassiotis and Kollias, 2001). TNFR-1 is required for noxious pro-inflammatory activities of TNF-α but not the immunosuppression (Kassiotis and Kollias, 2001). T_H_1 and T_H_17 cells are activated through the TNFR-1 pathway while Tregs are activated through the TNFR-2 pathway (Chen and Oppenheim, 2011). This suggests targeting the TNFR-1 pathway may be beneficial in MS. Indeed, treatment of mice with a TNFR-1 blocker reduced the severity and onset of EAE and supressed T_H_1 and T_H_17 responses (Nomura et al., 2011). TRAF2 knockouts skew towards T_H_17/T_H_1 responses so are not appropriate (Lin et al., 2011b). TRAF3 negatively regulates IL-17 receptor signaling and suppresses NF-κB activation (Zhu et al., 2010). TRAF3 binding to IL-17R prevents the IL-17R–Act1–TRAF6 signaling activation complex from forming (Zhu et al., 2010). Therefore, it is not surprising that TRAF3 attenuates EAE and thus may be a therapeutic option (Zhu et al., 2010). Dominant negative TRAF6 can block IL-17F-triggered ubiquitination of IL-17R, preventing downstream signaling (Rong et al., 2007).

It has been found that NIK-knockout mice are resistant to EAE (Jin et al., 2009) While T_H_17 differentiation is inhibited, NIK-deficient cells can commit to the other effector lineages and thus NIK inhibition is specific (Jin et al., 2009). Because NIK deficiency impairs T_H_17 development, all T_H_17-derived inflammatory factors are impaired, leading to total EAE resistance (Jin et al., 2009). NIK deficiency in T cells also impairs effector function, with reduced IFN-γ expression (Jin et al., 2009). A different study showed that NIK signaling is required for DC secretion of T cell-instructive cytokines IL-12/IL-23p40 and IL-16 (T_H_17 inducer), and its knockout caused EAE resistance (Hofmann et al., 2011).

Inhibitors of IKK itself are another option. The mechanisms include ATP analogs that have specificity for IKK, allosteric inhibitors of IKK and compounds that interact with an activation loop of IKKβ (Gupta et al., 2010). These inhibitors have a 200-fold preference for IKKβ compared with IKKα (Gupta et al., 2010). There is evidence supporting the potential of IKK inhibition with mice possessing inactive IKKα being refractory to EAE and having defective T_H_17 cell differentiation (Li et al., 2011). Similarly, IKKβ knockouts are completely resistant to EAE (van Loo et al., 2006; Greve et al., 2007) In addition, peptides that mimic the NEMO-binding domain of IKK proteins can inhibit NF-κB as well as hinder T cell effector function, leading to protection from EAE (Dasgupta et al., 2004). Treatment of mice with IKK-inhibitory compound, PS-1145, decreased disease severity when administered during the induction of the disease (Greve et al., 2007). Inhibition of IKKβ and NEMO in resident CNS cells interferes with NF-κB and ameliorates EAE in mice (van Loo et al., 2006).

Other inhibitors downstream of IKK include activators of protein phosphatases, proteasome inhibitors, IκB ubiquitination blockers, NF-κB nuclear translocation inhibitors, p65 acetylation inhibitors, methyltransferase inhibitors, DNA binding inhibitors, molecules that up-regulate IκB and IκB super-repressors (Gupta et al., 2010). Proteasome inhibitors such as bortezomib (PS-341) and PS-519 have been trialed in EAE. Both have been shown to inhibit NF-κB activation, reduce pro-inflammatory T cell response and ameliorate EAE (Vanderlugt et al., 2000; Fissolo et al., 2008). It has been shown that degradation of IκB, the inhibitor of RelA, is increased in EAE and by preventing degradation of IκB with TPCK (a protease inhibitor), the incidence of EAE was decreased, with less severe disease and quicker recovery (Hwang et al., 2011). A mutant version of IκB-α that is resistant to degradation has been shown to cause reduced effector responses in T cells after TCR stimulation (Aune et al., 1999). The frequency of a gene polymorphism of the IκB-α gene promoter (−708–8bpins) has been reported to be decreased in primary progressive MS (Miterski et al., 2002). PDTC, which directly blocks p50 and p65, inhibits NF-κB activation and pro-inflammatory gene expression in rat spinal cords and attenuates the clinical symptoms of EAE (Liu et al., 1999; Pahan and Schmid, 2000).

As for which subunit of NF-κB to target, there is evidence that c-Rel-deficient mice are resistant to EAE and that targeting RelA can also lessen EAE severity (Chen et al., 2011; Hwang et al., 2011). RelB signaling is more complicated as it can act as both an activator and repressor of NF-κB-dependent gene expression (Marienfeld et al., 2003). Some studies suggest RelB inhibits T_H_17 responses through epigenetic changes at the IL-17 locus that prevent RORγt binding and that RelB-deficient mice have exacerbated EAE (Xiao et al., 2015). This suggests a protective role for RelB in EAE. Another study has found that Malt1, an NF-κB regulator, is a critical determinant of the encephalitogenic potential of T_H_17 cells (Brüstle et al., 2012). Malt1 deficiency leads to a failure to cleave RelB, allowing it to act as a suppressor of canonical NF-κB signaling (Brüstle et al., 2012). Furthermore, Malt1-deficient mice have poor IL-17 and GM-CSF expression and are protected from EAE (Brüstle et al., 2012). However, others note that RelB is required for RORγt and RORα4 expression and T cell differentiation into γδT cells, a population that constitutively expresses the IL-23 receptor and produces IL-17, IL-21 and IL-22 in response to IL-1β and IL-23 (Sutton et al., 2009; Petermann et al., 2010; Powolny-Budnicka et al., 2011). This cell population increases susceptibility to EAE and has been found in increased levels in the brains of mice with EAE (Sutton et al., 2009). Therefore the targeting of RelB is not clear cut as of yet and a better understanding of its function is required.

p50 may be a reasonable target. NF-κB1-deficient mice are significantly resistant to EAE, possibly due to its roles in activation and differentiation of autoreactive T cells (Hilliard et al., 1999). A review speculates that the phenotype of NF-κB1-knockout mice is complicated by the effects that loss of p105 has on the stability of the proteins with which it associates (Beinke and Ley, 2004). TPL2 and ABIN-2 are severely reduced in p105-knockouts and thus the TPL2–MEK–ERK pathway is affected as well as the NF-κB pathway (Beinke and Ley, 2004).

While NF-κB inhibition is appealing, certain mice knock-out models such as RelA, IKK and NEMO, are embryonic lethal and c-Rel and RelB knockouts show impaired immunity (Beg et al., 1995; Köntgen et al., 1995; Weih et al., 1995; Li et al., 1999a,b; Liou et al., 1999; Kim et al., 2003; Schmidt-Supprian et al., 2004). The effects of various NF-κB knockout combinations have been reviewed extensively and will not be further elaborated here, but it is important to note that the concept of NF-κB inhibition is complex and a greater understanding of the role of each subunit and component of the NF-κB pathway is required in order to develop appropriate targets for inhibition without impairing cellular immunity and responses to infections (Gerondakis et al., 2006).

Other possible NF-κB inhibitors include: antioxidants; bacterial, fungal and viral proteins that inhibit NF-κB; anti-inflammatory and immunosuppressive agents; and p53 induction, which has been shown to repress NF-κB (Gupta et al., 2010).

Anti-inflammatory and immunosuppressive agents are currently used in MS treatment. EAE can take various forms, depending on the neuroantigen used to induce the EAE and the rodent strain used (Donia et al., 2010). Lewis rat EAE is monophasic and self-remitting, Swiss-Jackson Laboratories mice and Dark Agouti rats have a relapsing and remitting EAE and C57Bl/6 mice have a chronic progressive course (Donia et al., 2010). Dexamethasone administration prophylactically and in early disease effectively suppressed EAE development in all four of these groups (Donia et al., 2010). The mechanism for this may depend on NF-κB as its activation is hindered by dexamethasone treatment of macrophages (Crinelli et al., 2000). IκB-α gene transcription was increased and TNF-α expression decreased after dexamethasone treatment (Crinelli et al., 2000). Therefore, glucocorticoids induce IκB-α expression, preventing NF-κB activation and thus inhibiting cytokine gene expression (Auphan et al., 1995; Scheinman et al., 1995). Glucocorticoid receptors can also interact with NF-κB proteins, and interfere with their DNA binding (Ray and Prefontaine, 1994).

Interferon-β (IFN-β) treatment causes increased levels of the protein tyrosine phosphatase SHP-1 in PBMCs, which has a role in inhibiting cytokine signaling, pro-inflammatory gene expression and CNS demyelination (Christophi et al., 2009). IFN-β also decreases NF-κB and STAT6 activation while increasing STAT1 activation (Christophi et al., 2009). SHP-1 inhibits NF-κB expression (Neznanov et al., 2004). Increased SHP-1 has modest inhibitory effects on STAT1 activation that is overcome by the direct activating effect of IFN-β (Christophi et al., 2009). STAT1 expression is pro-inflammatory and could be responsible for the side effects of IFN-β (Christophi et al., 2009).

## New MS Therapies Related to NF-κB Signaling Pathways

Evidently, NF-κB plays a key role in MS development, through many different signaling pathways. Each signaling pathway has many influencers, some of which act in many of the NF-κB signaling pathways, and others that are limited to only a few NF-κB signaling pathways. Modulating NF-κB is clearly a reasonable target for therapeutic intervention. However, whether direct inhibition of NF-κB is the best mechanism remains to be elucidated. Inhibition of the influencers on NF-κB signaling may be a better approach as this can allow fine-tuning of NF-κB signaling and the potential targeting of dysfunctional proteins, restoring the NF-κB balance. Interestingly, four promising therapies, fingolimod, teriflunomide, dimethyl fumarate (DMF) and laquinimod (LAQ), all modulate NF-κB signaling in some way and with encouraging clinical evidence, three of the four have already gained FDA approval.

## Fingolimod

**Figure 6:**
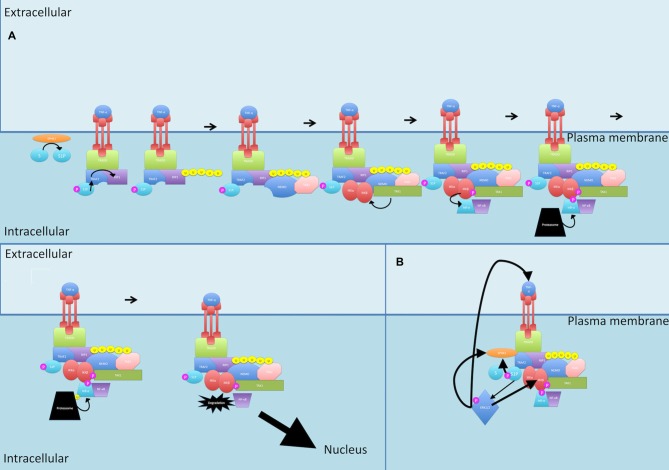
**Sphingosine signaling.** **(A)** During canonical signaling, when TRAF2 binds to the TNF-α receptor, it recruits SPHK1. SPHK1 catalyzes S1P formation. S1P then acts as a cofactor for TRAF2-mediated K63-linked polyubiquitination of RIP1. **(B)** S1P also activates ERK1/2 and these together further activate SPHK1. ERK1/2 activation leads to activation of IKK and increased TNF-α production, increasing total flow through the canonical pathway. P, phosphate; U, ubiquitin.

Fingolimod is a new oral therapy for MS. It is an analog of sphingosine, a molecule that has been implicated as an influencer of NF-κB signaling (see Figure 6). The starting point of the normal pathway is the activation of the kinase SPHK1 by numerous stimuli, notably pro-inflammatory cytokines such as TNF-α, PDGF, VEGF and EGF, which promote that translocation of SPHK1 to the plasma membrane where it phosphorylates sphingosine to form sphingosine-1 phosphate (S1P; Lebman and Spiegel, 2008; Colombo et al., 2014). S1P acts on the S1P receptors S1P1-S1P5 in an autocrine or paracrine manner (Spiegel and Milstien, 2011).

TNF-α is a potent activator of SPHK1 (Napolitano and Karin, 2010). When TRAF2 binds to the TNF-α receptor, it recruits SPHK1 to induce the catalyzation of S1P formation (Napolitano and Karin, 2010). Interestingly, S1P then acts as a cofactor for TRAF2 and is essential for the TRAF2-mediated K63-linked polyubiquitination of RIP1 (Napolitano and Karin, 2010).

S1P is also able to cause activation of ERK1/2 (Kim et al., 2000). Both ERK2 and S1P further activate SPHK1, with ERK2 phosphorylation of SPHK1 increasing its affinity for the plasma membrane (Takabe et al., 2008).

Putting this together, S1P is required for initiating the canonical signaling pathway at an upstream position (through TRAF2 and RIP), S1P activates ERK1/2 which leads to activation of IKK, an integral member of canonical signaling, and ERK1/2 also leads to TNF-α production, increasing total flow through the canonical pathway, culminating in increased NF-κB production.

Fingolimod, the sphingosine analog, is rapidly phosphorylated into its active form where it modulates S1P1, S1P3, S1P4 and S1P5 receptors (Colombo et al., 2014). However, unlike S1P, binding of fingolimod to the receptors causes their internalization and degradation, thereby halting the signaling processes that have been discussed and in turn, decreasing NF-κB production and TNF-α signaling (Colombo et al., 2014). Fingolimod also has been shown to attenuate IL-17 signaling which has been discussed as a key player in NF-κB production (Liao et al., 2007).

Fingolimod also affects lymphocyte migration. The S1P1 receptor is responsible for T cell migration and accumulation in peripheral lymph nodes (Yopp et al., 2005). In the presence of intact chemokine systems, T and B cells require S1P1 activity for egress from lymphoid organs (Yopp et al., 2005). S1P requires ABCB1 and ABCC1 multidrug lipid transports to access the extracellular space and activate the S1P1 receptor (Honig et al., 2003). CCR7 is expressed on T cells and its ligands, CCL21 and CCL19, in the T zone stromal cells. S1P1 acts on the lymphocytes to promote lymph node egress by overcoming retention signals mediated by CCR7 (Pham et al., 2008). CXCR4 and CCR2 have been found to follow the same pattern (Yopp et al., 2005).

The importance of the interference in sphingosine signaling is multifactorial. Peripherally, fingolimod prevents the trafficking of immune cells out of secondary lymphoid organs, thus preventing their migration into the CNS where the autoimmune attack on myelinated neurons occurs (Colombo et al., 2014). Fingolimod is also suspected to be able to cross the blood brain barrier and therefore can act centrally on astrocytes (Colombo et al., 2014). It has been shown that activation of the S1P pathway and the resultant NF-κB production activates astrocytes to sustain scar formation, and release toxic mediators that contribute to neuroinflammation and neurodegeneration, leading to formation of MS lesions (Colombo et al., 2014). Fingolimod has been shown to impair astrocyte activation and thereby block neurodegeneration induced by astrocytes (Colombo et al., 2014). It is hypothesized that fingolimod exerts a neuroprotective effect through this hampering of astrocyte responses but does not directly rescue neurons (Colombo et al., 2014). However, Bridela and Lalivea (2014) show that it may actually promote a reparative process in the CNS.

## Teriflunomide

Teriflunomide is an active metabolite of leflunomide (LN), which is an anti-rheumatic drug (Bridela and Lalivea, 2014). It functions by inhibiting dihydroorotate dehydrogenase, a key enzyme involved in the de novo pyrimidine synthesis pathway that is used heavily by rapidly proliferating lymphocytes (Bridela and Lalivea, 2014). This blockage leads to a cytostatic effect on proliferating B and T cells (Warnke et al., 2009).

Interestingly, a second effect has been studied (Manna and Aggarwal, 1999). A study found that LN blocked the TNF-α induction of NF-κB in a dose-dependent way (Manna and Aggarwal, 1999). The study surmised that LN blocks the activation of NF-κB by other stimulants too (Manna and Aggarwal, 1999). It was found that LN inhibits the TNF-induced p56lck and MEK activation (Manna and Aggarwal, 1999). P56lck is involved in the activation of ERK signaling and so is MEK, therefore, this drug also acts on the pathway discussed previously to decrease NF-κB production (Li et al., 2008).

Another study has found that at low concentrations, LN inhibits proliferation of T cells by inhibiting pyrimidine biosynthesis as it is reversed when uridine is replaced (Elder et al., 1997). However, at higher concentrations, uridine no longer reverses the inhibition of proliferation, suggesting another pathway is operating at this concentration (Elder et al., 1997). It is inferred that the mechanism at this dose is the inhibition of protein tyrosine kinases JAK1 and JAK3 as well as p56lck, as IL-2 induced phosphorylation of JAK1 and JAK3 are reduced at this concentration (Elder et al., 1997).

To understand this interaction, it is crucial to understand how signaling through IL-2 is initiated. First, as IL-2 receptor does not possess kinase activity, it must recruit JAK (Williams, 2011). The β chain of the receptor binds to JAK1, p56lck and STAT proteins (Williams, 2011). The ϒ chain binds to JAK3 (Williams, 2011). Heterodimerization bringing the β and ϒ chain into proximity causes phosphorylation of JAK1 and p56lck by JAK3 and initiates the binding of the STAT proteins (Williams, 2011). Activated p56lck then activates P13K and anti-apoptotic BCL-2 proteins (Williams, 2011). Thus an anti-apoptotic pathway is initiated (Williams, 2011). So it is clear that inhibition of p56lck or prevention of JAK3 phosphorylation would interfere with the proliferation and activation of lymphocytes as well as the production of cytokines (Warnke et al., 2009). It is important to remember that the effects of IL-2 are initiated by factors such as NF-κB so there is yet again interplay with this pathway.

Another effect of teriflunomide is an interference with the interaction between T cells and antigen-presenting cells (APC) crucial for T cell immune responses (Zeyda et al., 2005). Whether this is through the disturbance of cell adhesion molecules and matrix metalloproteinase is not known (Warnke et al., 2009).

## Dimethyl Fumarate

DMF is another therapy for treating MS. It acts through multiple mechanisms: it stimulates an anti-inflammatory response via a Nrf2-dependent antioxidant response pathway; it inhibits NF-κB signaling in an Nrf2-independent way, leading to decreased inflammatory cytokine production; and it interferes with maturation and function of antigen-presenting cells with deviation from T_H_1 and T_H_17 responses to a T_H_2 phenotype (Gerdes et al., 2007; Gold et al., 2012b; Peng et al., 2012; Linker and Gold, 2013; Gillard et al., 2015).

Reactive oxygen species are thought to play a role in MS pathogenesis. Reactive oxygen species activate NF-κB, leading to the production of cytokines such as TNF-α and IL-12 (Nicholas et al., 2014). The ability of MS patients to cope with oxidative stress appears to be diminished as evidence by decreased glutathione (GSH) in the CSF: GSH is a key detoxifier of free radicals and exogenous toxins (Peterson et al., 1998; Calabrese et al., 2003; Nicholas et al., 2014). It is theorized that low levels of oxidative stress lead to a cellular antioxidant response through Nrf2-mediated phase II enzyme expression (Kim et al., 2007). At higher levels of stress, MAPK and NF-κB are activated, exacerbating the pro-inflammatory effects (Kim et al., 2007). At the highest levels of stress, apoptosis occurs (Kim et al., 2007).

The internal GSH state in antigen presenting cells has been found to be a crucial regulator of the immune response (Kim et al., 2007). It has been found that when GSH levels are depleted, T_H_2 responses are favored. (Kim et al., 2007) This response is characterized by IL-4 and IL-10 as well as antibody responses and has a significant anti-inflammatory, repair-orientated effect (Peterson et al., 1998). In contrast, repletion of GSH skews the immune response towards that of a T_H_1 response, leading to IFN-γ and IL-12 production (Peterson et al., 1998; Kim et al., 2007). Leukocytes are especially sensitive to these GSH changes (Kim et al., 2007).

The importance of this phenomenon becomes clear when discussing DMF. This molecule induces initial GSH depletion, purportedly through formation of a stable complex (Schmidt and Dringen, 2010). The depletion in GSH leads to expression of hemoxygenase-1 (HO-1), which has been confirmed to be upregulated after administration of DMF (Ghoreschi et al., 2011). HO-1 acts as an anti-inflammatory and antioxidant molecule and protects cells by inhibiting several immune effector functions(Lehmann et al., 2007). One way in which HO-1 has been proposed to generate an anti-inflammatory milieu is through modulation of cytokine expression. HO-1 has been found to inhibit IL-12 and IFN-γ through DMF in immune cells (Lehmann et al., 2007). Additionally, upon activation, HO-1 is cleaved and translocates to the nucleus where it interacts with the IL-23p19 promoter in the NF-κB site and prevents transcription of IL-23 (Ghoreschi et al., 2011). Taken together, this provides evidence that HO-1 could mediate a shift from a T_H_1/T_H_17 response, favoring production of IL-12, IL-23 and IFN-γ, towards a T_H_2 response that generates Il-4, due to initiation by type 2 dendritic cells that produce IL-10 (Ghoreschi et al., 2011). Indeed studies show HO-1 plays a protective role in EAE and deficiency of HO-1 leads to an exaggerated inflammatory response (Nicholas et al., 2014). Interestingly, expression of HO-1 is reduced in PBMCs of MS patients, especially during disease exacerbation (Fagone et al., 2013). HO-1 produces carbon monoxide (CO) as a by-product of heme catabolism and thus is a carbon monoxide-releasing molecule (CORM; Fagone et al., 2012). CORMs can create a controlled quantity of CO which may be effective in treating diseases of immune dysregulation (Fagone et al., 2012). HO-1 has been shown to be protective in EAE models and CO administration had similar effects (Chora et al., 2007) Endogenous HO-1 and CO may be protective in EAE and MS and these could represent a novel therapeutic option for MS (Fagone et al., 2012).

In addition, DMF impairs STAT1 phosphorylation, thereby preventing IL-12p35 transcription. This was confirmed in another study, where DMF decreased production of IL-12 and Il-6 (Peng et al., 2012). IL-12 leads to IFN-γ producing CD4^+^ cells (T_H_1) while IL-6 without IL-12 leads to a T_H_17 differentiation (Lovett-Racke et al., 2011). This provides further evidence of the shift to the T_H_2 response and the induction of an anti-inflammatory, antioxidant environment (Ghoreschi et al., 2011).

DMF has been shown to increase nuclear levels of another antioxidant—Nrf2 (Lin et al., 2011a). Normally, Nrf2 is sequestered in the cytosol and polyubiquitinated by KEAP1, leading to constitutive degradation (Gillard et al., 2015). Oxidative stress changes the interaction between Nrf2 and KEAP1, leading to Nrf2 translocation to the nucleus where it can transcribe antioxidant and detoxification genes (Scannevin et al., 2012; Gillard et al., 2015). Related to this, DMF interacts with KEAP1 and results in the stabilization of Nrf2 and translocation to the nucleus where it can exert its antioxidant functions (Gillard et al., 2015). Some of the genes induced by Nrf2 are GSH-related enzymes, therefore, while there is an initial decrease in GSH concentration (after 2 h), the GSH concentration is increased after 24 h (Lin et al., 2011a).

Expression of MHC class II, CD80 and CD86 is also hampered by DMF, creating an immature dendritic cell phenotype that cannot activate IL-17- or IFN-γ-producing CD4^+^ T cells (Peng et al., 2012). Interestingly, it was also found that GSH depletion leads to inhibition of the LPS-induced expression of CD80, CD87 and CD54 expression on DCs which is under the control of NF-κB (Kim et al., 2007). In addition, it is known that NF-κB is required for the development of T_H_1 responses (Li and Verma, 2002). So, these findings raise the question of whether DMF interferes with NF-κB signaling and through this, impairs DC maturation and impairs T_H_1 responses.

DMF impairs NF-κB signaling through multiple mechanisms (Peng et al., 2012; Gillard et al., 2015). DMF suppresses ERK1/2 which has been discussed previously as an initiator of NF-κB signaling (Peng et al., 2012). DMF also suppresses MSK1 the kinase downstream of ERK1/2 which is involved in regulation of transcription. This is significant as MSK1 phosphorylates p65, thereby increasing transcriptional activity; first, by stabilizing p65, permitting nuclear localization; and second, by enhancing binding to coactivators and basal transcription factors (McCoy et al., 2005; Peng et al., 2012). MSK1 and NF-κB may form a transcription complex in inflammatory conditions, dependent on this phosphorylation by MSK1 and thus MSK1 is required for NF-κB activation and subsequent transcription (Reber et al., 2009). Therefore, an additional effect of DMF is the reduction of p65 nuclear localization through prevention of phosphorylation to stabilize the transcription factor (Peng et al., 2012). DMF may further prevent NF-κB translocation through the attenuation of IκB-α degradation (Lin et al., 2011a). Thus, DMF modulates NF-κB signaling mechanisms and, perhaps through hindering NF-κB, impacts DC maturation and cytokine production.

DMF has also been shown to inhibit TNF-α-induced expression of the adhesion molecules VCAM-1, ICAM-1 and E-selectin (Vandermeeren et al., 1997). Their expression relies on the activation of a cytokine-inducible enhancer in the promoter of their genes and these enhancers all contain a NF-κB responsive element (Vandermeeren et al., 1997). Indeed, it has been shown that inhibiting NF-κB blocks TNF-α induction of E-selectin, VCAM-1 and ICAM-1 (Read et al., 1995). Upregulation of these adhesion molecules on the endothelium, promotes migration of leukocytes from blood vessels into tissues (Vandermeeren et al., 1997). Therefore, DMF can prevent leukocyte influx into the brain tissue and decrease inflammation.

## Laquinimod

LAQ is a quinolone-3-carboxamide small molecule (Bridela and Lalivea, 2014). LAQ can diffuse across the blood brain barrier and so can target the CNS immunity (Bridela and Lalivea, 2014; Kieseier, 2014).

Studies show that NF-κB activation in astrocytes is involved in demyelination and that LAQ interferes with NF-κB activation to prevent demyelination (Brück et al., 2012). Many mechanisms have been proposed. One study showed that LAQ treatment slows down the degradation of IκB-α, leading to reduced nuclear translocation of p65 (Brück et al., 2012). A different group found that expression of IKKβ and NEMO was decreased after incubation with LAQ, leading to increased amount of IκB-α (Jolivel et al., 2013). Another study found increased gene expression of the NF-κB inhibitor NF-KBIE and suppression of BTRC which increases ubiquitination of the inhibitor (Gurevich et al., 2010).

Transcription of NF-κB-controlled genes, IL-1β, TNF-α, MIP1b, CXCL9 (a T_H_1 chemokine involved in T cell trafficking), LY9 (involved in lymphocyte activation) and ICAM (involved in activation and extravasation of leukocytes), were reduced after DC treatment with LAQ (Gurevich et al., 2010; Jolivel et al., 2013). In addition, degradation of p100 was also impaired by LAQ (Jolivel et al., 2013).

NF-κB is required for maturation of DCs, which in turn induces T cell differentiation (Jolivel et al., 2013). Thus, blockage of NF-κB by LAQ, creates an immature DC phenotype with reduced ability to induce CD4^+^ T cell proliferation, pro-inflammatory cytokine secretion, chemokine production and consequent migration of monocytes as well as a decrease in DC number (Haggiag et al., 2013). However, unlike the effects of DMF, where CD86 and CD80 were down-regulated, LAQ upregulates CD86 while not modulating CD80 (Jolivel et al., 2013). This upregulation of CD86 on its own is thought to promote a semi-mature differentiation-locked tolerogenic DC phenotype (Jolivel et al., 2013). Indeed CD86 and not CD80 is required for immune tolerance (Liu et al., 1999). Tolerogenic DCs induce T cell apoptosis, anergy and regulatory T cells (Tregs; Li and Shi, 2015). Not surprisingly then, it has also been found that LAQ augmented the Treg cell response leading to further anti-inflammatory effects (Haggiag et al., 2013). It is important to note that* ex vivo* studies did not replicate the increase in CD86 (Stasiolek et al., 2015).

LAQ has also been shown to reduce the levels of TNF-α, IFNα, CSCL10, IL-23p19 and IL-12 p35 while upregulating anti-inflammatory cytokines TGFβ, IL-10 and IL-4 (Gurevich et al., 2010; Brück et al., 2012; Kieseier, 2014). LAQ thereby modulates APCs to increase the occurrence of the anti-inflammatory type II monocyte, shifting from the pro-inflammatory T_H_1 phenotype (Brück and Wegner, 2011; Haggiag et al., 2013).

Another effect of LAQ is to reduce the entry of pro-inflammatory T cells into the CNS (Kieseier, 2014). The mechanism is by proposed lowering of MMP9 which regulates migration of monocytes into inflamed tissues (Kieseier, 2014). In addition, one study surmised that LAQ also led to down-regulation of VLA-4 which binds to VCAM1 (Kieseier, 2014). This is significant as VLA-4, expressed by leukocytes, mediates firm adhesion to activated endothelial cells of the blood-brain barrier, leading to transmigration and is a target of the drug natalizumab (Schwab et al., 2015). However, with natalizumab, there are questions regarding whether the benefit of the drug justifies the risk of progressive multifocal leukoencephalopathy, so perhaps LAQ may be a safer alternative (Schwab et al., 2015) The mechanism whereby this down-regulation occurs is not known, but a study has shown that IL-4 suppresses VLA-4 expression thus perhaps the ability of LAQ to induce T_H_2 cells and IL-4 production influences VLA-4 expression (Sasaki et al., 2009). However, a different study suggest that it is not down-regulation of VLA-4, but lack of responsiveness to CCL21, a chemokine trigger, that causes the decreased adhesiveness of VLA-4 (Wegner et al., 2010). The authors propose that it is the inhibitory effect of LAQ on IL-17 and IL-13, which normally increase VLA-4 responsiveness to CCL21, that is the mechanism (Wegner et al., 2010). Secretion of IL-17 is indeed decreased by LAQ (Brück and Wegner, 2011).

Constitutive nitric oxide synthase expression by astrocytes has been implicated in the development of MS plaques through the production of nitrite oxide and superoxide radicals, which can cause damage to oligodendrocytes, myelin sheaths and axons (Brück et al., 2012). Neuronal death in MS is mediated by NO, and MMP-9 can also kill neurons (Mishra et al., 2014). The JNK, AKT and 90RSK pathways can also generate neurotoxins (Mishra et al., 2014). This is important as LAQ treatment suppresses inducible STAT1, NOS, JNK, AKT, MMP and 90RSK, thereby preventing neurotoxin development (Schulze-Topphoff et al., 2012; Mishra et al., 2014). Clinically, in a mouse model of demyelination involving cuprizone-treated mice, LAQ prevented demyelination as well as microglia activation, axonal transections, reactive gliosis and oligondendroglial apoptosis (Brück et al., 2012). Therefore, it is proposed that LAQ might have a neuroprotective effect via microglia (Mishra et al., 2014).

## Clinical Data

Data from clinical trials comparing the new therapies to a placebo group can be seen in Table 1. The trials indicate that these drugs can significantly reduce the annualized relapse rate compared to placebo, with fingolimod offering the greatest reduction. These drugs can also halt disease progression, except for lower-dose teriflunomide, which recorded no reduction in progression. Fingolimod and LAQ were noted to protect from brain loss. In addition, fingolimod, teriflunomide and DMF outperformed LAQ in improvements in MRI indicators of disease progression.

**Table 1:** **Data from clinical trials of the new therapies for multiple sclerosis (MS)**.

Drug	Trial	Dose	% reduction in annualized relapse rate	% reduction in disease progression			MRI signs			Measure of tissue damage or loss		
				At 3 months	At 6 months	At 2 years	% reduction in gadolinium enhancing lesions per T1 scan	% reduction in new lesion on T2 at 2 years	New T1 lesions	% reduction in volume of lesions on T1	% reduction in volume of lesions on T2	Brain loss reduction at 2 years
Fingolimod	FREEDOMS	0.5 mg	54.0*	27.6*	34.2*	NA	81.0*	74.0*	NA	82.6*	68.6*	35.9*
	(Kappos et al., 2010)	1.25 mg	60.0*	31.1*	39.4	NA	81.0*	74.0*	NA	75.9*	95.2*	32.0*
Teriflunomide	TEMSO	7 mg	31.5*	20.5	NA	NA	57.1*	NA	NA	5.7	51.5*	25.0
	(O’Connor et al., 2011)	14 mg	31.5*	26.0*	NA	NA	80.5*	NA	NA	37.7*	76.7*	25.0
Teriflunomide	TOWERS	7 mg	22.0*	NA	NA	−7.1	NA	NA	NA	NA	NA	NA
	(Confavreux et al., 2014)	14 mg	36.0*	NA	NA	19.8*	NA	NA	NA	NA	NA	NA
Dimethyl fumarate	DEFINE	240 mg BD	52.7*	NA	NA	40.7*	94.4*	84.7*	NA	NA	NA	NA
	(Gold et al., 2012a)	240 mg TDS	47.2*	NA	NA	33.3*	72.2*	74.1	NA	NA	NA	NA
Dimethyl fumarate	CONFIRM	240 mg BD	45.0*	NA	NA	23.5	75.0*	70.7*	57.1*	NA	NA	NA
	(Fox et al., 2012)	240 mg TDS	50.0*	NA	NA	23.5	80.0*	73.0*	65.7*	NA	NA	NA
Laquinimod	ALLEGRO	0.6 mg	23.0*	NA	NA	29.3*	37.3*	29.6*	NA	NA	NA	33.1*
	(Comi et al., 2012)
Laquinimod	BRAVO	0.6 mg	17.6	23.1	30.0*	NA	21.4	16.5	NA	NA	NA	27.2*
	(Vollmer et al., 2014)

Data from seven clinical trials was extracted and % reductions in clinical indicators and MRI indicators compared to placebo were calculated and presented in the above table. *p < 0.05.

## Conclusion

It is clear that in MS, the pro-inflammatory NF-κB signaling pathways are out of balance. NF-κB contributes to MS pathogenesis and could represent a target for therapeutic intervention. The pre-clinical model, EAE, highlights the potential of molecules inhibiting elements of the NF-κB signaling pathway as therapeutic options. Furthermore, the four drugs discussed in this review all modulate the NF-κB pathway in some respect, albeit through different mechanisms. Researches have been expressing that targeting elements of this pathway is paramount to treating MS, but it is clear that systemic blockage of NF-κB would be unsafe as NF-κB has different effects in different cell types, some of which are protective, some detrimental (Kabashima et al., 2004; Mc Guire et al., 2013). NF-κB is also required for normal cell functions, and it is essential for mounting proper immune responses (Kabashima et al., 2004). Indeed, certain therapies that operate too far upstream of NF-κB may have disastrous consequences and affect related signaling pathways.

More targeted therapies will be required to redress the signaling faults. As far as which molecule should be the target, it is clear that MS is a multifactorial disease not dependent on only one gene, or inciting insult. It is entirely possible that there are a variety of aberrant genes present in MS patients that decrease the functioning of any of the many kinases and molecules implicated in the NF-κB signaling pathway. This begs the question as to whether patients be genetically screened for variants associated with NF-κB signaling, as this may identify patients amenable to NF-κB or cytokine blockade (Kabashima et al., 2004).

Therefore, crucial to the treatment of MS in the future will be individualized treatment, dependent on the fault in the signaling pathway. Improved knowledge of the specific activities of NF-κB and NF-κB regulatory mechanisms in controlling inflammatory and protective responses in these different cell types in MS and EAE is required to better understand the disease and possible therapeutic targets.

## Author Contributions

SML had substantial contributions to the conception or design of the work, drafting the work and final approval of the version to be published and agrees to be accountable for all aspects of the work in ensuring that questions related to the accuracy or integrity of any part of the work are appropriately investigated and resolved. JY had substantial contributions to the conception or design of the work, revising the work critically for important intellectual content, final approval of the version to be published and agrees to be accountable for all aspects of the work in ensuring that questions related to the accuracy or integrity of any part of the work are appropriately investigated and resolved.

## Funding

This work was supported by the Project Grant (10-024), Postdoctoral Fellowship (2003) from MS Research Australia and UQ Postdoctoral Fellowship (2003) for JY from the University of Queensland, Brisbane, Australia. We thank ANZgene consortium, Australia for supporting research material.

## Conflict of Interest Statement

The authors declare that the research was conducted in the absence of any commercial or financial relationships that could be construed as a potential conflict of interest.
